# Food Industry Byproducts in Confectionery: Functional Jelly Candy Development from Black Carrot Pomace and Olive Oil

**DOI:** 10.3390/foods14203524

**Published:** 2025-10-16

**Authors:** Süheyla İlgün Biltekin, Aysel Elik Demir, Hatice Neval Özbek, Fahrettin Göğüş

**Affiliations:** 1 Department of Food Engineering, Engineering Faculty, University of Gaziantep, Gaziantep 27310, Türkiye; 2 Department of Food Technology, Vocational School of Technical Sciences, Tarsus University, Mersin 33400, Türkiye

**Keywords:** functional jelly candy, black carrot pomace, virgin olive oil, stability

## Abstract

This study aimed to develop a functional jelly candy enriched with black carrot pomace (BCP) and virgin olive oil, promoting both nutritional enhancement and byproduct valorization. Three jelly formulations were prepared using BCP at levels of 6.7 g, 8.7 g, and 10.7 g, and apple juice concentrate (AJC) at 39.6 g, 37.6 g, and 35.6 g, respectively. Texture profile analysis showed that increasing BCP content significantly reduced jelly hardness from 31.87 ± 2.23 N to 21.24 ± 1.04 N, springiness from 0.817 ± 0.014 to 0.622 ± 0.018, cohesiveness from 0.539 ± 0.051 to 0.370 ± 0.015, and chewiness from 13.99 ± 0.83 to 4.90 ± 0.47. Sensory evaluation conducted with 20 panelists revealed that the formulation with intermediate BCP (Formula 2) achieved the highest overall acceptability score (7.40 ± 1.54) and lowest oiliness perception (6.25 ± 2.05). The jelly exhibited 17.68 ± 0.46% moisture, 30.4 ± 0.03% total sugar, 5.6 ± 0.2% dietary fiber, 4.85 ± 0.29% protein, and 25.34 ± 1.44% fat content. Total phenolic content (TPC) was 178.76 ± 0.51 mg GAE/g dry basis, with a DPPH radical scavenging activity of 49.20 ± 5.66%. Encapsulation of olive oil within the jelly matrix reduced oxidative degradation over 50 days of storage, with peroxide values rising only from 5.00 to 6.00 meq/kg and acidity from 0.36% to 0.50%, compared to 6.25 meq/kg and 0.55% in free oil. These findings demonstrate that BCP-enriched jelly candies offer enhanced nutritional, functional, and oxidative stability properties while contributing to sustainable utilization of food industry byproducts.

## 1. Introduction

Jelly candies are widely consumed across all age groups and are traditionally formulated with gelling agents—such as gelatin, starch, pectin, or gellan—combined with sucrose, glucose syrup, acids, colorants, and flavorings to achieve their characteristic texture and taste [1,2]. In recent years, the jelly market has evolved to meet the growing demand for healthier and more functional products, including low-calorie, vegan, and nutrient-enriched variants fortified with bioactives such as vitamins, probiotics, and lipids [3,4]. This trend reflects a shift in consumer preferences toward health-conscious diets and presents opportunities for incorporating natural extracts and food byproducts with added nutritional value.

Recent studies have explored the enrichment of jelly formulations with various functional ingredients, including sage by-product extract [5], fish processing waste [6], black carrot liquid waste [7], unripe carob pods [8], and watermelon rind powder [9]. These approaches not only enhance the nutritional profile of jellies but also support waste valorization and sustainable food innovation.

At a broader scale, international reports have highlighted the need to reduce food processing waste to promote sustainable food systems. The UNEP Food Waste Index Report [10] estimates that a significant share of the 1052 million tonnes of global food waste in 2022 originates from the processing and production stages. Complementary resources such as the FAO Food Loss and Waste Database [11] and GRI 306: Waste [12] provide tools for tracking and mitigating industrial waste, while the UN Sustainable Development Goals (SDGs) call for urgent action to minimize food waste and enhance circular economy practices [13].

Black carrot pomace (BCP), a byproduct of juice and concentrate production, is rich in anthocyanins, phenolic compounds, and dietary fiber [14,15]. These compounds provide antioxidant activity and contribute to the product’s natural deep purple color and fruity aroma, offering a clean-label alternative to synthetic additives [16]. Virgin olive oil is another valuable ingredient, known for its high content of monounsaturated fatty acids and polyphenols that confer cardiovascular, anti-inflammatory, and antioxidant benefits [17,18]. However, its direct consumption can be limited by factors such as strong flavor, oxidation susceptibility, or inconvenience in daily use. Encapsulating olive oil in gel matrices offers a practical solution, enhancing its oxidative stability, masking undesirable sensory notes, and enabling convenient delivery through portioned, ready-to-eat formats like jelly candies [19,20]. The combined use of BCP and olive oil in jelly formulations has not been previously reported, and this approach offers a dual functional advantage: BCP contributes natural pigments, fiber, and antioxidants, while olive oil enriches the lipid profile with bioactive fatty acids and phenolic compounds. Their integration thus creates a multifunctional confectionery product that aligns with consumer demand for both health benefits and natural ingredients.

Therefore, this study aimed to develop a functional jelly candy enriched with BCP and virgin olive oil by incorporating food industry byproducts to enhance both nutritional quality and sustainability. Specifically, the effects of different levels of BCP and apple juice concentrate (AJC) on the textural, sensory, and compositional characteristics of the jelly were evaluated. Additionally, the encapsulation of olive oil was assessed for its impact on oxidative stability during storage. This approach intends to produce a clean-label, health-promoting confectionery with extended shelf-life and waste valorization potential.

## 2. Results and Discussion

### 2.1. Texture Profile Analysis Results

Table 1 presents the mean values and standard deviations for the mechanical properties—hardness, springiness, cohesiveness, and chewiness—of jelly formulations containing varying amounts of BCP and AJC.

Hardness is defined as the maximum force needed to cause an object to deform or change shape during the first bite [2], decreased significantly with increasing BCP content. Formula 1, which had the highest AJC and lowest BCP, exhibited the greatest hardness (31.87 ± 2.23 N), while Formula 3, with the highest BCP content, showed the lowest value (21.24 ± 1.04 N). This trend is consistent with the literature suggesting that fiber-rich ingredients weaken the gel network, leading to a softer texture [21]. Additionally, the sugar composition plays a crucial role in determining the texture of gummy and jelly-type candies, significantly influencing their hardness and stickiness [22]. Higher levels of sucrose and glucose led to an increase in the jelly’s hardness [4,23]. Formula 1, with the most apple juice concentrate, resulted in the highest hardness. The significant differences between the formulations (*p* < 0.05) indicate that the variation in ingredient proportions has a measurable impact on the texture. Sun, Xing, Qiu and Xiong [23] also reported that an increase in sugar concentration led to higher hardness values, which supports the findings of the present study.

Springiness, which refers to the sample’s ability to return to its original shape after deformation [2]. Formula 1 had the highest springiness (0.817), while Formulas 2 and 3 exhibited significantly lower values (0.738 and 0.622, respectively). This can be attributed to the higher sugar content in Formula 1, which likely enhanced the elasticity of the gel through the formation of a stable, hydrogen-bonded network [24].

Cohesiveness measures the extent to which a material changes shape under mechanical stress and is related to the internal structure’s strength and the resistance to breaking internal bonds [25]. Cohesiveness also followed a decreasing trend, with Formula 1 having the highest cohesiveness (0.539), followed by Formula 2 (0.427), and Formula 3 (0.370). The BCP fibers might interfere with the gelatin network, leading to a less cohesive matrix, making the jelly less uniform and more prone to breaking apart [26]. Cohesiveness value decreased with the addition of agricultural waste extract, similar to the findings of Kaynarca, et al. [27] study.

Chewiness, calculated by the product of springiness, cohesiveness, and hardness [28], indicates the energy required to chew the jelly until it is ready to swallow [29]. Chewiness values decreased significantly with increasing BCP levels, from 13.99 in Formula 1 to 4.90 in Formula 3. This is due to the combined reduction in hardness and cohesiveness in the high-BCP samples. Similar findings have been reported in studies involving grape seed or skin powder incorporation in soft candies [30].

In summary, increasing the proportion of BCP in jelly formulations results in a softer, less elastic, and less cohesive texture. Among the three, Formula 2 appears to strike a balance between textural firmness and softness, making it a promising candidate for consumer preference, which is further supported by sensory data (Section 2.2).

### 2.2. Sensory Analysis Results

The jelly samples were prepared following three different formulations: Formula 1, Formula 2, and Formula 3. For the sensory analysis, these samples were coded as 745 for Formula 1, 341 for Formula 2, and 958 for Formula 3. The sensory evaluation results of the jelly samples are presented in Table 2, based on a 9-point hedonic scale. A one-way ANOVA was conducted to statistically analyze the panelists’ scores for each sensory attribute. To determine which specific samples differed significantly, post hoc comparisons were performed using Tukey’s test.

**Figure 1:**
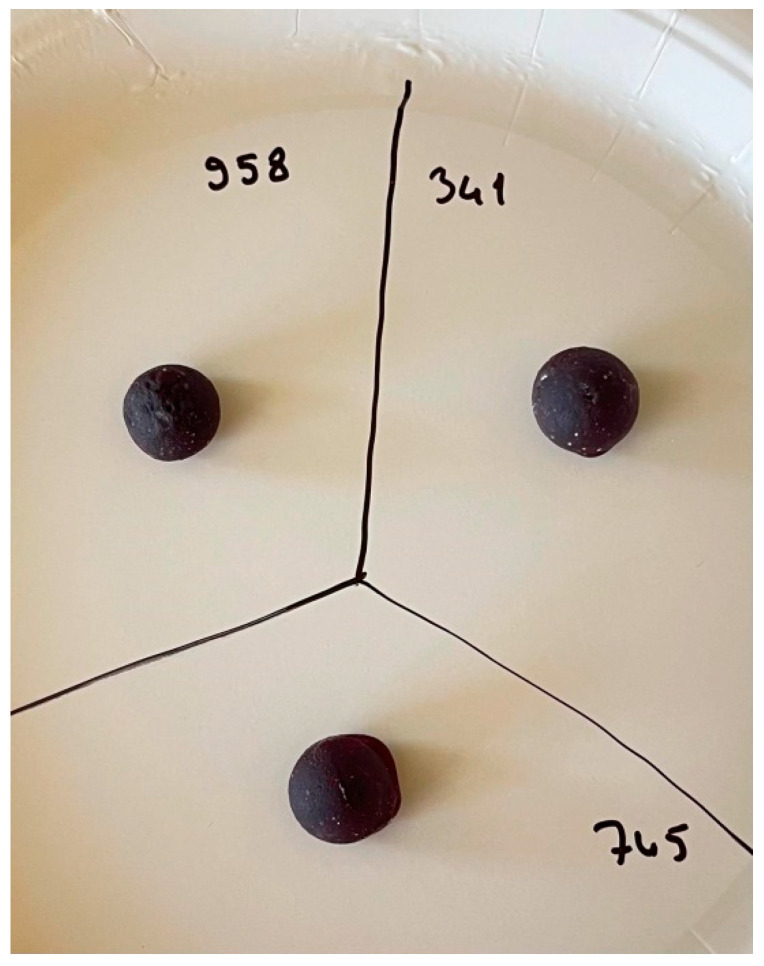
Jelly candy images for different formulations.

Appearance scores did not differ significantly among the samples (*p* > 0.05), with values ranging from 6.70 to 7.00. All three formulations exhibited comparable visual appeal, indicating consistent color and shape across the jellies. Figure 1 presents the image of the jelly candies produced with different formulations. The jelly candy has a cylindrical shape, with a diameter of 1.5 ± 0.1 cm, a height of 1.4 ± 0.1 cm, and a weight of about 2.5 ± 0.1 g.

The hardness scores reveal a stark contrast among the samples. Formula 1 and Formula 3 exhibited higher hardness scores than Formula 2, while there was no significant difference between the hardness scores of Formula 1 and 3. The results indicated that instrumental and sensory evaluations of hardness were not fully aligned. Instrumental analysis provides an objective measurement of the mechanical hardness of jelly samples, whereas sensory evaluation reflects the multidimensional perception experienced by panelists during oral processing. Therefore, differences between the two methods may occur. While instrumental measurements primarily capture parameters such as the force required for compression or fracture, panelists perceive hardness in combination with attributes such as elasticity, adhesiveness, disintegration, and dissolution in the mouth. As a result, a formulation identified instrumentally as softer may be perceived as harder in sensory evaluation. Moreover, test conditions—including probe type and compression speed—do not fully replicate the complex environment of the oral cavity, and thus discrepancies between instrumental and sensory results may arise. These findings emphasize that instrumental texture analysis and sensory evaluation are complementary, and a complete understanding of consumer perception requires consideration of both physical measurements and human sensory responses.

However, instrumental and sensory measurements do not always align. Texture is a multifaceted characteristic that encompasses the sensory attributes perceived during the handling (such as spreading and touching) and consumption of food [31]. The differences between texture profile analysis (TPA) and sensory evaluation are often more pronounced in soft products due to their unique textural behavior. While TPA provides objective and reproducible data, it may not accurately replicate how soft foods are compressed between the tongue and palate during consumption. In contrast, sensory analysis is based on human perception, which varies between individuals and is affected by complex interactions during mastication. These factors make it difficult to correlate TPA data with sensory results, particularly for soft-textured products [32]. In this study, discrepancies were observed between instrumental and sensory findings, which may be attributed to the panelists’ limited sensitivity in detecting the specific textural attributes of this particular product [33].

Formula 2 exhibited the highest chewiness score (4.60 ± 2.18), followed by Formula 3 (4.35 ± 2.87) and then Formula 1 (3.45 ± 2.14). The differences among the formulations were not statistically significant (*p* > 0.05). This suggests that panelists did not perceive a strong distinction in chewiness across the samples. These findings highlight that chewiness was not a distinguishing factor in consumer preference for these jelly formulations, and all samples were considered similar in terms of this textural property.

All three samples showed similar sweetness levels, with scores ranging from 4.45 ± 1.82 to 4.55 ± 1.79, and no statistically significant differences were observed (*p* > 0.05). This suggests that the formulations were well-balanced in terms of sweetness—an important factor for consumer acceptance. Interestingly, despite the varying amounts of AJC used across the formulations, sweetness perception remained consistent. This is particularly noteworthy, as apple juice concentrate, while a natural sweetener, still contributes free sugars. Therefore, its reduced use allows for the development of healthier formulations without compromising sweetness.

A similar trend was observed for sourness. Sourness scores ranged from 5.05 ± 1.70 in Formula 1 to 5.75 ± 1.71 in Formula 2, again with no significant differences between the samples (*p* > 0.05). This consistency suggests that the formulations likely contained similar types or levels of acidifying agents, resulting in a uniform sourness profile.

In this study, the sensory evaluation of jelly candies containing different concentrations of BCP and AJC showed no statistically significant differences in perceived fruitiness among the three formulations (*p* > 0.05). This result was somewhat unexpected, given that both BCP and AJC are rich sources of natural fruit-derived aroma compounds. One possible explanation lies in the high volatility and thermal sensitivity of these natural flavor compounds, which are prone to degradation during heat-intensive processes such as jelly preparation [1]. Additionally, the gel matrix itself may have played a role in suppressing aroma perception. According to Kim, et al. [34] hydrocolloid-based gels can entrap volatile aroma molecules within their polymer network, thereby limiting their migration from the interior of the gel to the surface, where they would otherwise be detected by sensory receptors. This physical entrapment could have contributed to the uniform fruitiness perception despite differences in the levels of BCP and AJC.

The sensory evaluation of oiliness in the jelly samples was conducted using a 9-point hedonic scale, where a score of 1 indicated a strong oily taste and 9 represented the absence of any oil perception. Statistical analysis using Tukey’s HSD test revealed significant differences among the three formulations (*p* < 0.05). Formula 2 received the highest oiliness score (6.25 ± 2.05), indicating that panelists perceived it as the least oily. In contrast, Formulas 1 (4.15 ± 2.32) and 3 (4.00 ± 2.79) were rated significantly lower, reflecting a stronger perception of oiliness. However, no significant difference was found between Formulas 1 and 3 (*p* > 0.05), suggesting a similar sensory response between the two. These findings imply that Formula 2 was more successful in masking the oil taste, potentially due to its lower concentration of black carrot pomace (BCP) and a more balanced level of apple juice concentrate (AJC). In comparison, the slightly higher oiliness perception in Formula 3 may be attributed to its greater BCP content, which could have interacted differently with the oil phase or affected the matrix structure.

The perception of oiliness (or fattiness) in emulsion-filled gels is influenced not only by oil concentration but also by the structural characteristics of the gel matrix. Studies have shown that when oil droplets are strongly integrated into the gel network, their release is minimized; however, increased oil content still results in a heightened perception of fattiness [35]. This suggests that fat perception is not solely dependent on free oil release, but also on the interaction between oil droplets and the gel matrix. The matrix’s ability to retain or release oil plays a crucial role in modulating this sensory attribute. Therefore, optimizing matrix composition is essential for controlling oiliness perception in gelled emulsions such as functional jelly candies.

Formula 2 received the highest acceptability score (7.40 ± 1.54), significantly outperforming Formulas 1 (5.70 ± 2.32) and 3 (5.60 ± 2.19) (*p* < 0.05), indicating that consumers found it more favorable. This preference is likely driven by Formula 2’s superior chewiness, balanced flavor, texture, and appearance, which together enhanced its overall sensory appeal.

Based on sensory and texture profile analyses, Formula 2 (Sample 341) demonstrated the most desirable attributes and emerged as the preferred formulation among panelists. Its reduced perception of oiliness, along with a slightly softer and less cohesive texture—reflected in intermediate hardness and chewiness values—contributed to its higher acceptability compared to Formulas 1 (Sample 745) and 3 (Sample 958).

While Formula 1 exhibited a firmer and more elastic texture, and Formula 3 was the softest, their stronger oiliness and lower chewiness negatively affected consumer preference. Other sensory attributes such as appearance, sweetness, sourness, and fruitiness showed no significant differences, suggesting they were not key factors in overall preference.

In conclusion, the balanced texture and reduced oiliness of Formula 2, likely resulting from adjustments in BCP and AJC, improved its sensory and texture characteristics, making it the optimal choice for consumer acceptance.

### 2.3. Chemical Properties of Jelly Candy

The properties of the jelly candy (Formula 2) are presented in Table 3. Jelly candies typically have a low moisture content ranging from 10 to 20%, which contributes to their characteristic texture and stability [22,36]. The sample in this study fits within this range with a moisture content of 17.68 ± 0.46%, close to the upper limit. This elevated moisture level can be attributed to the high water-holding capacity of pectin derived from black carrot pomace and gelatin used in its formulation. Moreover, the jelly’s relatively high sugar content (30.4%) likely aids moisture retention, as sugars effectively bind water and reduce evaporation [28,37].

The 25% oil content obtained in the black carrot jelly formulation after Soxhlet extraction can be attributed to the high proportion of olive oil included in the recipe, which makes up 35.29% of the formulation. However, the lower oil percentage compared to the formulation can be explained by the jelly matrix. Gelatin and pectin are key gelling agents widely used in jelly candies to achieve the desired texture and consistency. In the jelly formulation, gelatin contributes to elasticity and chewiness, which are characteristic properties of soft candies. Pectin, found in black carrot pomace, provides additional firmness and stability to the candy matrix. Together, these gelling agents form a cohesive gel structure by trapping water and other ingredients, creating the characteristic soft and bouncy texture of jelly candies [1].

Black carrot pomace provided a natural red–purple coloration to the jelly, eliminating the need for artificial additives while contributing anthocyanins and other phenolic compounds that enhance antioxidant capacity. The total phenolic content of the black carrot pomace-enriched jelly was 178 mg GAE/100 g dw, confirming a substantial presence of bioactive compounds. Comparative findings indicate that grape pomace-based jellies contain 115–156 mg GAE/100 g oven-dried pomace powder [38], apple pomace jellies reach much higher levels of ~825 mg/100 g dw [39], and pomegranate juice jellies show ~159 mg/100 g dw [40]. In this context, the TPC of black carrot pomace jelly was slightly higher than grape pomace and pomegranate juice jellies but lower than apple pomace jellies. These results demonstrate that the type of fruit byproduct strongly influences the phenolic profile of jelly products. Overall, incorporating black carrot pomace not only supports a clean-label approach through natural coloring but also provides functional and environmental value by enriching phenolic compounds, offering health benefits, and promoting sustainability through byproduct utilization.

The DPPH radical scavenging activity of the jelly was measured at 49.2%, indicating a moderate yet meaningful antioxidant capacity. This activity reflects the presence of bioactive compounds—primarily anthocyanins and other phenolics—originating from black carrot pomace. The ability to neutralize free radicals contributes not only to the jelly’s antioxidant profile but also enhances its nutritional value, aligning with growing consumer demand for functional foods offering health benefits beyond basic nutrition [41]. For comparison, a study on jelly formulated with grape pomace reported DPPH scavenging activities ranging from 29.9% to 65.8%, depending on the composition [38].

In the current jelly formulation, AJC was used as a natural sugar source, offering both sweetness and functional benefits without the need for artificial additives or sugar syrup. The total sugar content, measured at 30%, played a key role in enhancing the jelly’s texture, stability, and overall sweetness. Similar to molasses in gummy products, the high sugar content of AJC acts as a natural preservative, improving structural integrity and extending shelf life by reducing the need for synthetic additives [42]. Moreover, the use of AJC supports clean-label product development and aligns with consumer demand for natural ingredients. Beyond its preservative function, the natural sugars in AJC contribute to a balanced sweetness and desirable mouthfeel without introducing any undesirable flavor notes.

The jelly formulated with black carrot pomace contains 5.6% dietary fiber, qualifying it as a “source of fiber” according to European Regulation (EC) No 1924/2006, which requires products to contain between 3 g and 6 g of fiber per 100 g to carry this claim [43,44]. The incorporation of black carrot pomace not only enhances the nutritional profile of the jelly but also supports health-focused labeling aligned with regulatory standards and consumer expectations for functional foods.

The protein content of the black carrot jelly was measured at 4.85%, which is comparable to the 4.04% reported by Teixeira-Lemos, et al. [45] in an orange-based jelly formulated with gelatin and agar-agar. In the present study, 4% gelatin was incorporated, and the observed protein level suggests that both gelatin and black carrot pomace (BCP) contributed to the total protein content. Notably, BCP has been reported to contain 7.30% protein on a dry basis [15], further supporting its role in enhancing the nutritional value of the jelly formulation.

The ash content of the jelly was determined to be 0.28 ± 0.04%, reflecting the overall mineral composition of the ingredients used. This low value is consistent with the typical mineral profiles of BCP, apple juice concentrate, and gelatin, which are not inherently rich in minerals. Although BCP does contain fiber and other nutrients, its limited inclusion in the formulation results in a minimal contribution to ash content. This outcome aligns with the general expectations for jelly candies, which are primarily developed for their texture, flavor, and functional benefits rather than as mineral-rich products.

### 2.4. Monitoring Chemical Stability During Storage

To assess the chemical stability of the jelly formulations during storage, peroxide value (PV) and acidity content were monitored over a 50-day period under ambient conditions. These parameters are key indicators of lipid oxidation and hydrolytic degradation in oil-containing food systems.

Samples were stored in sealed containers at 25 ± 2 °C, protected from light, and analyses were conducted on days 0, 5, 10, 15, 20, 30, 40, and 50. Virgin olive oil and oil extracted from jelly samples were evaluated comparatively.

**Figure 2:**
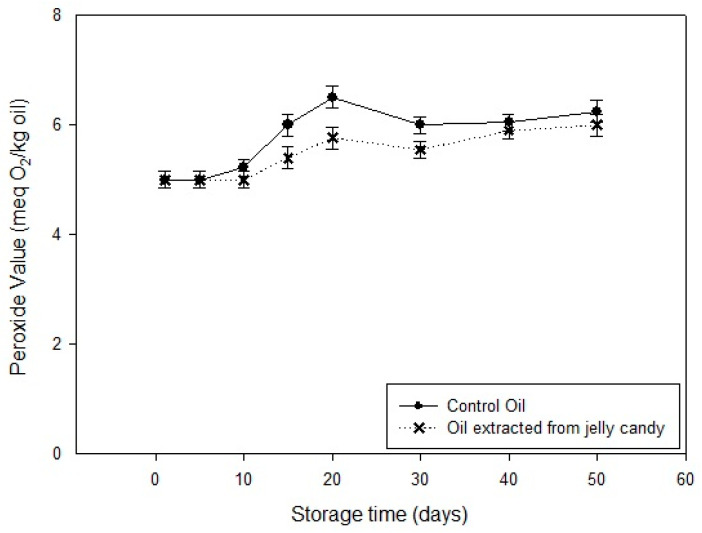
Change of peroxide value during storage.

As shown in Figure 2, PV values in virgin olive oil increased steadily from 5.00 to 6.25 meq/kg by day 50. In contrast, jelly-extracted oil exhibited a slower increase, reaching only 6.00 meq/kg. This suggests that the jelly matrix acted as a barrier to oxygen diffusion, slowing down oxidative reactions. A similar result has been reported in a previous study where gelled emulsion matrices limited lipid oxidation due to physical entrapment [46].

**Figure 3:**
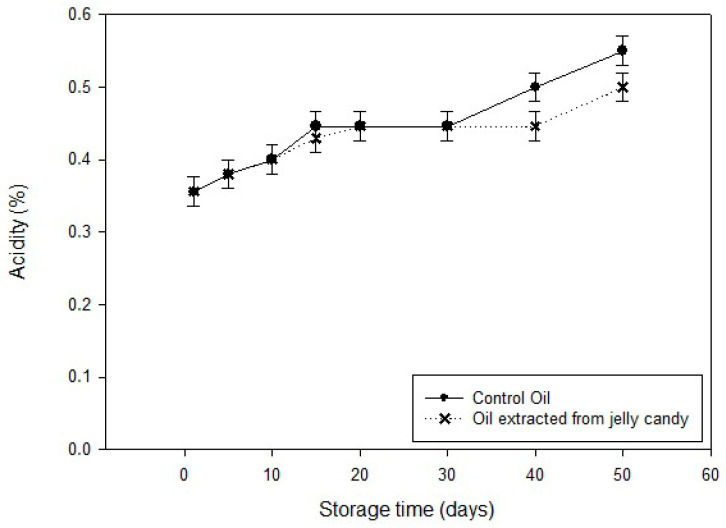
Change of acidity during storage.

Likewise, acidity levels in virgin olive oil rose from 0.356% to 0.550%, whereas in the jelly samples, they only reached 0.500% (Figure 3). The relatively lower increase in jelly-extracted oil indicates reduced hydrolytic degradation, likely due to decreased exposure to moisture and enzymes—an effect attributed to the protective role of the gel matrix and black carrot pomace.

In conclusion, the use of a gelatin-based matrix enriched with black carrot pomace effectively slowed both oxidative and hydrolytic degradation in olive oil during storage. This highlights the potential of food byproducts in extending shelf life and enhancing the nutritional quality of functional foods.

## 3. Materials and Methods

### 3.1. Materials and Jelly Preparation

BCP was taken from a producer of black carrot concentrate (Erkon Konsantre Co.) in Konya, Türkiye. The pomace was kept in sealed polyethylene bags at −70 °C until processing. Before treatment, BCP was equilibrated to room temperature overnight. Virgin olive oil was supplied from a local market (Gaziantep, Türkiye). AJC was supplied by Tasargem Enj. Inş. Mak. Müh. Gid. San. Tic. Lt. Şti., Adana, Türkiye. Gelatin supplied from Sel Sanayi Ürünleri Ticaret ve Pazarlama A.Ş., Istanbul, Türkiye. All chemicals used in this study were of analytical grade and supplied by Sigma Aldrich.

Initially, BCP was dried at 105 °C for 3 h then it was used in the jelly formulations. The moisture content of the pomace was 5.3 ± 0.04%. AJC, citric acid and 22.35 mL water were added to a beaker. The mixture was heated to 90 °C while stirring. BCP and xanthan gum were mixed and slowly added to the beaker. The mixture was cooked until it reached 105–107 °C. When the mixture became solid, the heat was turned off, and the temperature of the mixture was reduced to 90 °C. The gelatin and 7.65 mL water (at 50–55 °C) were mixed in another beaker and poured on to the mixture. The mixture was stirred quickly and the temperature was reduced to 60 °C. Virgin olive oil was then slowly added while stirring at a slow-fast speed until it became homogeneous. The mixture was poured into starch molds when it reached 40 °C and allowed to cool at room temperature. Afterwards, it was stored in the refrigerator at 4 °C. The formulations were presented in Table 4.

### 3.2. Methods

#### 3.2.1. Texture Profile Analysis

Texture profile analysis tests were performed using a TA.XT Plus Texture Analyzer (Texture Technologies Corp., Scarsdale, NY, USA/Stable MicroSystems, Godalming, UK) to determine hardness (N), springiness, cohesiveness, gumminess, chewiness, and resilience. A cylindrical compression probe with a diameter of 36 mm was used for the analysis. Stress area is 1017 mm^2^. The jelly samples have 0.75 cm height and 3.10 cm diameter.

#### 3.2.2. Sensory Analysis

A subjective sensory analysis was conducted by 20 trained panelists (students and lecturers from the Food Engineering Department of Gaziantep University). Panelists evaluated the jelly samples using a 9-point hedonic scale for 8 parameters: appearance, hardness, chewiness, sweetness, sourness, fruitiness, oiliness and acceptability. Samples were served at room temperature (25 °C).

#### 3.2.3. Moisture Content, Ash Content, Protein Content, Total Dietary Fibre

The ash content of the jelly was determined following the AOCS [47]. The moisture content of jelly was determined using vacuum drying at 70 °C until a constant weight was achieved. The protein content of the jelly was determined using the Kjeldahl Method. Initially, 1 jelly of the pectin sample was weighed into digestion flasks containing boiling chips. Then, 7 g of potassium sulphate and 0.01 g of copper sulphate were added, followed by 12.0 mL of sulphuric acid. The digestion flasks were placed into the digestion unit set at 400 °C for 40 min. After digestion, the flasks were transferred to the distillation apparatus (Kjeltec 2200 Auto Distillation Unit, Salford, UK) for distillation. The resulting distillate was titrated with 0.1 N HCl, and the volume used was recorded. Finally, the protein content of the pectin sample was calculated using a conversion factor of 5.55.

Total dietary fibre content of jelly was analyzed according to AOAC method 991.43 [47].

#### 3.2.4. Total Phenolic Content (TPC) by Folin-Ciocalteu’s Assay

The total phenol levels in the samples were assessed using a modified Folin-Ciocalteu method, adapted from Singleton, et al. [48].

#### 3.2.5. DPPH• Radical Scavenging Activity Assay

The antioxidant activity of the jelly was determined using the 2,2-diphenyl-1-picrylhydrazyl (DPPH•) radical scavenging assay, following the method outlined by Brand-Williams et al. [49]. Firstly, two grams of jelly were mixed with 20 mL of 80:20 methanol:water solution and stirred for 1 h to prepare the extract. The extract was then filtered. 0.1 mL of extract was combined with 3.9 mL of a 6 × 10^−5^ mol/L methanolic DPPH• solution. The mixture was then stirred and left to incubate for 30 min. After incubation, the absorbance of each solution was recorded at 515 nm, with pure methanol serving as the blank, using a spectrophotometer (Pharmacia Biotech Novaspec^®^ II, Cambridge, UK). The percentage of DPPH• radical scavenging activity was calculated using the following formula:

DPPH Radical Scavenging Activity (%) = (A_control − A_sample)/A_control × 100

#### 3.2.6. Total Sugar Content

Total sugars were extracted from each jelly by mixing with water for 30 min. Following filtration and analysis using the 3,5-dinitrosalicylic acid (DNS) method, as described by Özbek, et al. [50]. In brief, 1 mL of each sample was placed in test tubes, and hydrolysis was performed using 0.05 mL of concentrated HCl at 90 °C for 10 min. The solution was then neutralized with 0.15 mL of 5 N KOH, followed by the addition of 3 mL DNS reagent. The tubes were heated in a boiling water bath for 5 min, cooled to room temperature, and diluted to a total volume of 10 mL with distilled water. Absorbance was measured at 540 nm using a spectrophotometer (Optima SP 3000 Nano, Tokyo, Japan). Glucose was used to construct a calibration curve, and the results were expressed as g/100 g dry weight (dw).

#### 3.2.7. The Oil Content

For oil content determination, 10 g of jelly was first weighed and vacuum-dried at 70 °C for 2 h. The oil content was then measured using the Soxhlet extraction method. Oil from the samples was extracted using 125 mL of hexane in a macro automatic Soxhlet system (Gerhardt, type SE-416, Königswinter, Germany), and the oil was collected in an extraction cup. The extracted oil was weighed to determine the oil content. All experiments were performed in triplicate.

### 3.3. Monitoring Chemical Stability During Storage

Samples consisting of virgin olive oil and jelly were stored in amber glass containers specifically selected to prevent light-induced oxidation. The impermeable nature of glass effectively shields the samples from external environmental influences, while the amber coloration provides additional protection by filtering out light wavelengths known to accelerate oxidation [51]. To further reduce oxidative degradation, the containers were flushed with nitrogen gas to displace oxygen and establish an inert atmosphere. This combination of container material, color, and inert gas flushing ensured optimal preservation conditions during the storage period.

The 50-day storage study was conducted at room temperature to mimic typical storage environments. Sampling was performed systematically, with the first samples collected at five-day intervals in the early period to capture early changes, followed by sampling every ten days to monitor longer-term trends. This protocol enabled comprehensive tracking of acidity and peroxide value variations in both jelly-encapsulated and control olive oil samples, allowing thorough evaluation of oxidative-chemical stability throughout the experiment.

#### Peroxide Value and Acidity

The oil was extracted from the jelly using a cold extraction method [52]. For this, the jelly was weighed, and hexane was added at a solid-to-liquid ratio of 1:10. The mixture was shaken for 1 h at room temperature (25 °C). After shaking, the solvent mixture was filtered through filter paper, and the free oil was collected by evaporating the hexane under vacuum.

Peroxide value (PV) was measured following a modified version of the method outlined by Elik, et al. [53] and expressed in milliequivalents (meq) of O_2_ per kilogram of oil. Approximately 0.5 g (m) of the extracted oil was placed in a 100 mL Erlenmeyer flask, to which 10 mL of chloroform and 15 mL of acetic acid were added. Then, 1 mL of saturated potassium iodide was introduced, and the mixture was shaken for 1 min. The flask was left in the dark for 8 min. Afterward, 25 mL of distilled water and 1 mL of a 1% starch indicator solution were added. The solution was titrated with 0.01 N sodium thiosulfate (Na_2_S_2_O_3_) until the endpoint was reached. The volumes of Na_2_S_2_O_3_ consumed during the sample titration (a) and used in the blank titration (b) were recorded in mL. All measurements were performed in triplicate. The peroxide value was calculated using the following equation.

PV ((meq O_2)/(kg oil)) = ((a − b) × N × 1000)/m

The acidity of the extracted oil was measured according to ISO 660 [54]. An equal volume of 100 mL of ethanol and diethyl ether was mixed, and 2 mL of phenolphthalein was added as an indicator. The mixture was then titrated with 0.1 M NaOH until a permanent light pink color was obtained, indicating that the alcohol was neutralized. Next, 1 g of extracted oil was weighed, and the neutralized alcohol was added, followed by another titration with 0.1 M NaOH until the pink color appeared again. The same procedure was repeated for a blank titration. The calculations were performed using the formula below and expressed as a percentage of oleic acid.

Acidity = (V c M × 100)/(1000 × m)

V is the volume, in millilitres, of the standard volumetric sodium hydroxide solution used; c is the concentration, in moles per litre, of the standard volumetric sodium hydroxide solution used; M is the molar mass, in grams per mole, of the acid chosen for expression of the result, 282 for oleic acid; m is the mass, in grams, of the test portion.

### 3.4. Statistical Analysis

One-way ANOVA was used to analyze the data obtained from the sensory evaluation. The data underwent statistical analysis via SPSS statistical software, version 22.0 (SPSS Inc., Chicago, IL, USA). Each analysis was repeated three times and averaged. Results are presented as means ± standard deviation (SD), with significant differences (*p* < 0.05) between means being identified.

## 4. Conclusions

This study developed a clean-label jelly candy enriched with BCP and encapsulated virgin olive oil, showing potential as a functional confectionery. The incorporation of BCP significantly influenced textural properties by reducing hardness, springiness, cohesiveness, and chewiness. Among the three formulations, the sample with intermediate BCP content (Formula 2) demonstrated the highest overall sensory acceptability and lowest perceived oiliness, indicating optimal consumer appeal.

Nutritional analysis revealed that the jelly provided 5.6% dietary fiber and 4.85% protein, while delivering 178.76 mg GAE/g total phenolics and 49.20% DPPH radical scavenging activity. Encapsulation of olive oil within the jelly matrix effectively minimized oxidative degradation during 50 days of storage, with lower increases in peroxide value and acidity compared to free oil.

Overall, the study indicates the potential of BCP and olive oil encapsulation as a sustainable and health-oriented approach in confectionery. This formulation strategy is in line with clean-label trends and may contribute to food industry byproduct valorization, suggesting a promising direction for the development of nutritionally enhanced jelly products.

## Figures and Tables

**Table 1:** Texture profile analysis of jelly.

	Formula 1	Formula 2	Formula 3
Hardness (N)	31.871 ± 2.230 ^a^	25.630 ± 4.413 ^b^	21.240 ± 1.035 ^c^
Springiness	0.817 ± 0.014 ^a^	0.738 ± 0.007 ^b^	0.622 ± 0.018 ^c^
Cohesiveness	0.539 ± 0.507 ^a^	0.427 ± 0.031 ^b^	0.370 ± 0.015 ^c^
Chewiness	13.985 ± 0.834 ^a^	8.028 ± 0.914 ^b^	4.900 ± 0.474 ^c^

^abc^ Different letters within a row indicate significant differences (*p* < 0.05).

**Table 2:** Sensory analysis results.

	Formula 1 (745)	Formula 2 (341)	Formula 3 (958)
Appearance	6.70 ± 1.92 ^a^	6.80 ± 1.67 ^a^	7.00 ± 1.80 ^a^
Hardness	7.35 ± 1.46 ^a^	3.15 ± 2.23 ^b^	7.05 ± 1.73 ^a^
Chewiness	3.45 ± 2.14 ^a^	4.60 ± 2.18 ^a^	4.35 ± 2.87 ^a^
Sweetness	4.50 ± 1.88 ^a^	4.55 ± 1.79 ^a^	4.45 ± 1.82 ^a^
Sourness	5.05 ± 1.70 ^a^	5.75 ± 1.71 ^a^	5.20 ± 2.09 ^a^
Fruitiness	5.30 ± 2.13 ^a^	5.75 ± 1.62 ^a^	5.55 ± 2.14 ^a^
Oiliness	4.15 ± 2.32 ^a^	6.25 ± 2.05 ^b^	4.00 ± 2.79 ^a^
Acceptability	5.70 ± 2.32 ^a^	7.40 ± 1.54 ^b^	5.60 ± 2.19 ^a^

^ab^ Different letters within a row indicate significant differences (*p* < 0.05).

**Table 3:** Chemical properties of jelly candy for Formula 2.

Properties	Values
Moisture content (%, w.b.)	17.68 ± 0.46
Dietary fiber (%, w.b.)	5.6 ± 0.2
Protein (%, w.b.)	4.85 ± 0.29
Sugar (%, w.b.)	30.4 ± 0.03
Ash (%, w.b.)	0.28 ± 0.04
Fat (%, w.b.)	25.34 ± 1.44
TPC (mg GAE/g DW)	178.76 ± 0.51
DPPH (%)	49.20 ± 5.66

**Table 4:** Jelly formulation.

Materials	Formula 1		Formula 2		Formula 3	
	(g)	(%)	(g)	(%)	(g)	(%)
Gelatin	5.10	4.00	5.10	4.00	5.10	4.00
AJC	39.60	31.06	37.60	29.49	35.60	27.92
Water	30.00	23.53	30.00	23.53	30.00	23.53
BCP	6.70	5.25	8.70	6.82	10.70	8.39
Citric acid	1.00	0.78	1.00	0.78	1.00	0.78
Xantham gum	0.10	0.08	0.10	0.08	0.10	0.08
Virgin olive oil	45.00	35.29	45.00	35.29	45.00	35.29
**Total**	127.5	100 *	127.5	100 *	127.5	100 *

* Percentages may not add up to exactly 100% due to rounding.

## Data Availability

The raw data supporting the conclusions of this article will be made available by the authors on request.

## Abbreviations

The following abbreviations are used in this manuscript:

BCP	Black carrot pomace
AJC	Apple juice concentrate
PV	Peroxide value
TPC	Total phenolic content
